# Archival mitogenomes identify invasion by the *Batrachochytrium dendrobatidis* CAPE lineage caused an African amphibian extinction in the wild

**DOI:** 10.1098/rspb.2024.1157

**Published:** 2024-07-31

**Authors:** Thomas R. Sewell, Lucy van Dorp, Pria N. Ghosh, Claudia Wierzbicki, Christian Caroe, John V. Lyakurwa, Elena Tonelli, Andrew E. Bowkett, Stuart Marsden, Andrew A. Cunningham, Trenton W. J. Garner, Tom P. Gilbert, David Moyer, Ché Weldon, Matthew C. Fisher

**Affiliations:** ^1^ Department of Infectious Disease Epidemiology, MRC Centre for Global Infectious Disease Analysis, White City, Imperial, London W12 0BZ, UK; ^2^ Department of Genetics, Evolution & Environment, UCL Genetics Institute, University College London, London WC1E 6BT, UK; ^3^ Institute of Zoology, Zoological Society of London, London NW1 4RY, UK; ^4^ Section for Evolutionary Genomics, The GLOBE Institute, University of Copenhagen, Øster Voldgade 5-7, Copenhagen 1353, Denmark; ^5^ Department of Zoology and Wildlife Conservation, University of Dar es Salaam, P.O. Box 35064, Dar es Salaam, Tanzania; ^6^ Department of Natural Sciences, Manchester Metropolitan University, Manchester M1 5GD, UK; ^7^ Wild Planet Trust, Paignton Zoo, Totnes Road, Paignton TQ4 7EU, UK; ^8^ Unit for Environmental Sciences and Management, North-West University, Potchefstroom, South Africa; ^9^ Integrated Research Center, Field Museum of Natural History, Chicago, IL, USA

**Keywords:** amphibian, extinction, habitat change, chytridiomycosis, emerging infectious disease, *Batrachochytrium dendrobatidis*

## Abstract

Outbreaks of emerging infectious diseases are influenced by local biotic and abiotic factors, with host declines occurring when conditions favour the pathogen. Deterioration in the population of the micro-endemic Tanzanian Kihansi spray toad (*Nectophrynoides asperginis*) occurred after the construction of a hydropower dam, implicating habitat modification in this species decline. Population recovery followed habitat augmentation; however, a subsequent outbreak of chytridiomycosis caused by *Batrachochytrium dendrobatidis* (*Bd*) led to the spray toad's extinction in the wild. We show using spatiotemporal surveillance and mitogenome assembly of *Bd* from archived toad mortalities that the outbreak was caused by invasion of the *Bd*CAPE lineage and not the panzootic lineage *Bd*GPL. Molecular dating reveals an emergence of *Bd*CAPE across southern Africa overlapping with the timing of the spray toad's extinction. That our post-outbreak surveillance of co-occurring amphibian species in the Udzungwa Mountains shows widespread infection by *Bd*CAPE yet no signs of ill-health or decline suggests these other species can tolerate *Bd* when environments are stable. We conclude that, despite transient success in mitigating the impact caused by dams’ construction, invasion by *Bd*CAPE caused the ultimate die-off that led to the extinction of the Kihansi spray toad.

## 1. Introduction

Worldwide, amphibian populations are in steep decline and being increasingly threatened by anthropogenic habitat alteration [1,2], climate change [3,4] and the spread of infectious disease [5–7]. Three decades of research on the disease chytridiomycosis and its causative pathogens, chytrid fungi in the genus *Batrachochytrium* and specifically *Batrachochytrium dendrobatidis* (*Bd*), has solidified the proximate link between these emerging infections and global amphibian declines [6–8]. The epidemiology of these multi-host pathogens of ectothermic amphibians are extraordinarily complex with diverse biotic and abiotic factors known to modify the outcome of host–pathogen interaction post invasion [9]. These factors include the suite of environmental variables that comprise the host amphibians fundamental ecological niche [10,11] as well as host susceptibility [12] and the genotype of *Bd* [13]. Accordingly, multiple lines of evidence are needed to establish causal associations between *Bd* infection and species declines or extinctions. However, owing to the intrinsic difficulty of working with wildlife alongside the understudied nature of many wildlife diseases, the causal epidemiological inference is usually conducted *post hoc* and is qualitative rather than quantitative [7]. This problem is compounded by the need to rely on the confirmation of amphibian chytridiomycosis along with *Bd* DNA in archived museum specimens [14,15]. Previously, the molecular detection of *Bd* from archived specimens has relied on either quantitative PCR or ribosomal ITS-2 metabarcoding, neither of which provides reliable phylogenetic information on pathogen genotype [13,16]. However, an expanding constellation of molecular techniques are increasingly available for the multi-locus analysis of microbes from museum specimens [17], and we here develop a shotgun sequencing method that is agnostic to *Bd* lineage as well as appropriate for phylodynamic inference.

**Figure 1.**
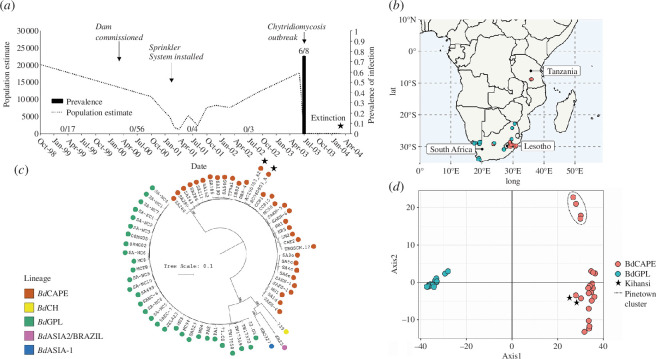
Time series of the Kihansi gorge epizootic and determination of outbreak *Bd*CAPE lineage. (*a*) Time-series of the Kihansi epizootic showing changes in the population size of *N. asperginis* alongside the prevalence of *Bd* and key events (data from [18,20,21]). (*b*) Map of southern Africa indicating the location of *Bd*CAPE and *Bd*GPL isolates considered for ML genetic clustering. (*c*) A midpoint rooted ML tree constructed from 1205 high-quality single nucleotide polymorphisms (SNPs) within the 178 kbp mitochondrial genome. The phylogeny was generated using RAxML utilizing the GTR model and CAT rate approximation. The two Kihansi archival samples (AC040803_A2 and AC290703_A2) are highlighted with stars. The five major lineages of *Bd* are denoted by tip colour with good bootstrap support between divergence events. (*d*) Principal component analysis (PC 1–2) based on mitochondrial SNPs (45 individuals, 724 SNPs), generated from sequence of cultured South African *Bd* isolates plus the two Kihansi archival samples (stars); the South Africa Pinetown cluster is also shown. Colours are based on predicted clustering generated using snapclust in R, which fully resolved both *Bd*GPL and *Bd*CAPE lineages. CAT, category; GTR, generalized time reversible; ML, maximum likelihood.

A striking example of an amphibian species extinction is that of the Kihansi spray toad (*Nectophrynoides asperginis*), an ovoviviparous bufonid, once micro-endemic to the Kihansi Gorge in the Udzungwa Mountains of Tanzania [18,19]. This species had a spatially restricted population within the spray wetlands associated with waterfalls in the gorge [18]. In 2000, the toads’ habitat experienced a 10-fold reduction in water flow owing to the construction of the Lower Kihansi Hydropower Project, following which the spray toad population sharply declined to fewer than 2000 individuals by March 2001 (figure 1*a*) [18,22]. Conservation measures to restore the wetland’s ecologically important habitat initially appeared successful with moderate habitat regeneration and rapid recovery to almost 18 000 toads by June 2003 (electronic supplementary material, figure S1) [18,21]. However, the spray toad population entered a catastrophic and enigmatic decline soon after the 2003 survey and in 2009, the species was officially declared extinct in the wild [19]. Factors connected to the Lower Kihansi Hydroelectric Project, such as a modification of wetland size and composition, ineffective mitigation measures, and the accidental release of contaminated sediments, were considered principal drivers of habitat alteration and the consequent decline-to-extinction of *N. asperginis* [18,23].

Evidence of *Bd* infection in *N. asperginis* and histopathology that identified acute lethal clinical chytridiomycosis with chytrid thalli covering >50% of the skin surface was recorded during the terminal decline of the wild population (electronic supplementary material, figures S2 and S3). *Post hoc* examination of archived spray toads, collected from the Kihansi Gorge between 1996 and 2002, was used to establish that *Bd* was absent until their ultimate decline during June 2003–February 2004. These data include 99 *N*. *asperginis* also collected from the Kihansi Gorge between 1996 and 2002 and 400 toads exported for *ex situ* captive breeding efforts in the United States of America [20]. Taken together, these observations led to the conclusion that invasion by *Bd* caused the extinction event in this micro-endemic species, comprising the first recorded host extinction caused by this pathogen in Africa [20]. To better understand the epidemiology of *Bd* in the extinction event, we accessed the last two preserved *N. asperginis* field-collected cadavers that had been collected at the time of the 2003 population crash. Using this archived fixed tissue, we applied sequencing protocols developed for the analysis of ancient DNA to shotgun sequence samples for genomic epidemiological analysis of the outbreak. Alongside these molecular investigations, we describe retrospective and more recent surveillance of co-occurring amphibian species in the Udzungwa Mountains to determine the extent to which *Bd* has invaded populations and species in the region.

## 2. Material and methods

### (a) Library preparation and sequencing of Kihansi spray toad samples

Four ethanol preserved specimens of *N. asperginis* were provided by Ché Weldon of North-West University. The spray toads were originally collected dead from the Kihansi Gorge in the Udzungwa Mountains, Tanzania (−8.575, 35.851389) (electronic supplementary material, figures S1–S3) between June and August 2003; this was during the spray toads terminal population decline [20]. The samples were kept in ethanol until use.

One foot from each Kihansi spray toad was removed and air dried for 2 h. DNA was extracted using Qiagen Dneasy Blood and Tissue Kit according to the manufacturer instructions, including the recommended double elution step to maximize DNA yield. Subsequently, libraries were built using the Nebnext Ultra II FS library kit (New England Biolabs, Ipswich, MA, USA) with an average target fragment length of 300–700 bp. Adaptor-ligated libraries were purified with 1× volume of SPRI beads [24]. qPCR was carried out to estimate amplification cycle numbers for the indexing PCR. Libraries were subsequently amplified using NEBs Q5 2× mastermix for 10 cycles. Amplified libraries were purified with SPRI beads as before and purified libraries were analysed on an Agilent 2100 Bioanalyzer. Pooled samples were sequenced on two lanes on an Illumina HiSeq 4000 instrument in single read mode for 80 cycles. DNA extractions were undertaken in the ancient DNA clean facility at the The GLOBE Institute, University of Copenhagen.

### (b) Read alignment and variant identification

Genome Analysis Toolkit (GATK) v. 3.6 was used to call variant and reference nucleotides from two subsets of Bd mitochondrial alignments; firstly, a collection of previously sequenced Bd isolates representing five deeply diverged *Bd*-lineages for phylogenetic analysis; secondly, a novel collection of WGS *Bd*GPL and *Bd*CAPE isolates collected in South Africa for de novo genetic clustering. The two Kihansi samples, AC040803_A and AC290703_A2, were included in each set of alignments for comparison. All sequenced reads were trimmed using cutadapt v 1.9.1 to remove adapter sequences and low-quality ends (Phred score <20). BWA v. 0.7.8 was used to align the trimmed reads to the both the nuclear and mitochondrial reference assembly of the Bd isolate Jel423 (Broad Institute), and samtools v. 1.3.1 was used to generate mapping statistics. Mitochondrial alignments were selected for subsequent analysis owing to their percentage breadth of coverage. Prior to variant calling, alignments were pre-processed using AddOrReplaceReadGroup, to assign all reads per file to a single new read-group tag, and MarkDuplicates to identify duplicate reads. Variants were detected using HaplotypeCaller (GATK) in joint calling mode and with ploidy set to one. SelectVariants (GATK) was used to generate a SNP-only VCF file, and VariantFiltration was used to filter for high-quality variants (DP < 4 || MQ < 40.0 || QD < 2.0 || QUAL < 50).

During the analysis, we controlled for low read depth by using a joint variant calling approach (GATK Best Practices) which used previously sequenced *Bd* isolates representing the known global diversity of this pathogen (electronic supplementary material, table S1) [13]. This approach allowed us to leverage information from lineage-specific variants across a cohort of global isolates sequenced at a much greater depth, and in doing so providing us with increased confidence in variants that were called at a low depth in the two Kihansi samples.

Samples AC040803_A and AC290703_A2 generated 252 950 676 and 294 104 776 Illumina sequencing reads, respectively; of which, 28 745 (AC040803_A) and 15 343 (AC290703_A2) reads mapped successfully to the *Bd* mitochondrial reference assembly of isolate JEL423 (electronic supplementary material, table S2). Average depth of coverage when aligned to the reference mitochondrial assembly was 8.97× and 4.65×, respectively (electronic supplementary material, figure S4). At a depth of >2`, the AC040803_A alignment had a total of 1 60 904 bases covered (92.41%) and the AC290703_A2 alignment had 1 42 867 bases covered (82.05%). The average allele balance across both samples was 0.98, suggesting a single genotype associated with each infected toad.

### (c) Phylogenetic and population genetic analysis

SNPs generated from the subset of samples used for phylogenetic analysis were concatenated into a multi-sample FASTA and converted into PHYLIP format. As the variants were identified simultaneously across all samples using joint calling mode in GATK (where genotype calls are generated at every site where any sample in the call set has evidence for variation), a representative curated alignment of 1205 mitochondrial nucleotides was used for phylogenetic inference. A phylogenetic tree was generated using RAxML v. 8.2.9, employing the generalized time reversible (GTR) model and category (CAT) rate approximation. Phylogenetic uncertainty was assessed using 500 bootstrap iterations implemented via RAxML’s rapid bootstrapping mode. The consensus phylogeny was imported into R (v. 4.3.1) and visualized with associated bootstrap support using ggtree (v. 1.16.6) [25].

A multi-sample VCF file (725 SNP variants), generated from the subset of samples used for maximum-likelihood (ML) genetic clustering, was imported into R using vcfR [26] and analysed using the snapclust function in the adegenet package [27]. As prior lineage association of samples was known for all isolates excluding the two Kihansi spray toad samples, snapclust was supervised to discriminate two clusters (*k* = 2; *Bd*-GPL and *Bd*-Cape). The Ward clustering algorithm was applied for group membership. Principal component analysis (PCA) of mitochondrial variants was used to evaluate the clustering of isolates, which were coloured according to the group membership output generated by snapclust.

### (d) Bayesian dating of *Bd*CAPE diversity in Africa and tMRCA estimation

Bayesian phylogenetic tip-dating was conducted in BEAST2 (v. 2.6.3) , using an alignment of 30 *Bd*CAPE isolates with recorded sampling years between 2008 and 2017, plus the two Kihansi archival samples that were collected in 2003 (a final alignment of 32 individuals and 674 mitochondrial variants). Clustering analysis of African *Bd*CAPE isolates revealed a two-cluster split (figure 1*d*), with four isolates from Pinetown, South Africa forming their own separate cluster away from the remaining *Bd*CAPE genotypes. To assess the SNPs underlying this clustering, we ran Gubbins (v. 2.3.4) and Phandango (v. 1.3.0) [28,29] together with manual inspection of the alignment. Three putatively recombinant blocks were identified (A–C), distributed across all four Pinetown isolates and confirmed following visual check of the alignment (electronic supplementary material, figure S6). Removal of these regions of high SNP density resulted in a final alignment of 195 mitochondrial variants. A ML phylogeny was generated using iqtree (v. 1.6.12) with 100 bootstrap values to observe the effect pruning had on the resulting ML tree topology. A reduction in the length of the terminal branches was observed in the Pinetown cluster and the placement of isolate SA4c within the diversity observed among other *Bd*CAPE strains. The roottotip() function in BactDating was applied to identify the presence of a significant root-to-tip regression which was assessed for significance following 1000 random permutations of sampling dates [30]. Phylostems was also applied to visualize the extent of temporal correlation at each node in the phylogeny [31]. Both tools supported a measurably evolving alignment, justifying application of phylogenetic tip-calibration using all the southern African *Bd*CAPE isolates in our collection.

Using BEAST2, we initially applied a model averaging approach via the package bModelTest (v. 1.2.1) to identify the best supported site model for the dataset [32,33]. The two models with highest posterior support were 121324-25% posterior support (TN93) and 123423-22.97% posterior support (TIM). Given there was no strong preference for these or any other substitution models we proceeded with a model averaging approach. We then applied both relaxed and strict clock models across one of four possible demographic priors: Coalescent Constant Population, Coalescent Exponential Population, Coalescent Bayesian Skyline and Coalescent Extended Bayesian Skyline. BEAUti (v. 2.6.3) was used throughout to generate the xml input files. To ensure an appropriate rate calculation, the xml files were manually edited to include all the counts of invariant A, T, C & G sites. The MCMC chain length for all runs was set to 5 × 10^8^ and the trace log was set to record every 1000 samples, with convergence confirmed by an Effective Sampling Space >200 for all parameters and visual inspection of the MCMC traces. In addition, all models were run without the data—sampling from the prior—to confirm the posteriors estimated were not being driven by the choice of priors. To select the most appropriate clock and demographic model combination for our data, we applied the nested sampling function implemented in the NS package (v. 1.0.3) [34]. Bayes Factors (BFs) were generated through pairwise comparison of the estimate marginal likelihoods and interpretation of the BF ranges was made with accordance to Kass & Raftery [35] (electronic supplementary material, table S3B). All log output files were processed in R using the beastio and bdskytools packages (https://github.com/laduplessis) with the first 10% of the chain discarded as burn-in. Posterior distributions for each model were plotted using ggplot2^47^. To summarize the tree topology, a maximum clade credibility tree was generated using Tree Annotator (v. 2.6.3) specifying a 10% burn in percentage and considering the median value of node heights. The time calibrated phylogeny was visualized and annotated using ggtree in R.

**Figure 2.**
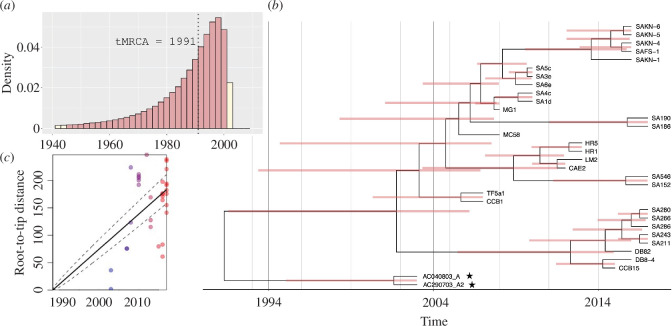
Bayesian tip-dating analysis of *Bd*CAPE mitochondrial diversity in Africa and with node calibrations estimated using BEAST2. The mean mitochondrial substitution rate was 2 × 10^−5^ substitutions per site per year [95% HPD, 6 × 10^−6^ to 3.5 × 10^−5^]. (*a*) Posterior distribution for tMRCA (tree height). Red bars indicate 95% HPD intervals of 14 and 67 years since most recent sampling date of 2017. Dotted black line indicates the mean tMRCA of sampled diversity to 1991 (26 years since most recent sampling date of 2017). (*b*) Maximum clade credibility phylogeny estimated using median node heights; Red bars indicate the 95% HPD for nodes. The two Kihansi archival samples are highlighted with a star. (*c*) Evidence of significant root-to-tip evolutionary divergence in mitogenomes for time-stamped isolates of *Bd.*

To assess the timing of emergence of *Bd*CAPE, we first confirmed the presence of significant temporal signal in the alignment (electronic supplementary material, figure S5) once confounding SNPs in four Pinetown isolates were excluded (electronic supplementary material, figure S6). We identified a positive correlation between root-to-tip phylogenetic distance and time of sampling which was significant following date randomization (figure 2*c*; electronic supplementary material, figure S2A–C). Subsequently, Bayesian tip-dating was applied in BEAST2 [32], testing various plausible demographic and clock models run until model convergence occurred (electronic supplementary material, table S3A). Following nested path sampling, the model with the highest marginal likelihood was found to be a relaxed clock with a coalescent constant population prior (electronic supplementary material, table S3B). The mean mitochondrial substitution rate was estimated as 2 × 10^−5^ substitutions per site per year [95% highest posterior density (HPD), 6 × 10^−6^–3.5 × 10^−5^] (electronic supplementary material, figure S7A). This rate was marginally faster than that previously estimated for the *Bd*GPL mitolineages, at 1.01 × 10^−6^ substitutions per site per year (10). Our analysis supports a recent time to most recent common ancestor (tMRCA) of sampled diversity to between 14 and 67 years ago (mean of 26 years since the most recent sampling date), pointing to an African introduction of *BdCAPE* between 1944 and 2003, with tMRCA of 1991 (figure 2).

### (e) Pan-lineage *Bd* diagnostic

Owing to a need to conserve DNA available for shotgun sequencing, Kihansi spray toad DNA extracts were not tested with the pan-lineage *Bd* diagnostic [36] but moved straight onto a lineage-specific qPCR assay [16], that distinguishes between *Bd*CAPE and *Bd*GPL lineages. Two of the four archived samples that showed the highest number of *Bd* genomic equivalent (GE) score, AC040803_A and AC290703_A2, were then shotgun-sequenced to confirm the lineage diagnosis and generate genotypic data for downstream analyses. Swab samples collected in the Udzungwa Mountains, Tanzania were processed according to the pan-lineage *Bd* qPCR diagnostic [36], except for 2005 samples (Survey no. 1, 2004–2005) that were shipped to Pisces Lab, USA for qPCR analysis. For 2013–2015 samples, swabs that had a >1 GE, were then processed using the lineage-specific qPCR assay [16].

Subsequently, Bayesian phylogenetic tip-dating was conducted in BEAST2 (v. 2.6.3) (28), using an alignment of 30 *Bd*CAPE isolates with recorded sampling years between 2008 and 2017, plus the two Kihansi archival samples that were collected in 2003 (a final alignment of 32 individuals and 674 mitochondrial variants) as described. Analyses supported a measurably evolving alignment, justifying application of phylogenetic tip-calibration using all the southern African *Bd*CAPE isolates in our collection.

### (f) Swab sample surveys of Udzungwa Mountain amphibians

Swab sampling of amphibians for *Bd* infection was undertaken across the Udzungwa Mountains for two time periods: Survey no. 1, 2004–2005; and Survey no. 2, 2013–2015.

#### (i) Survey no. 1, 2004–2005

Amphibians were collected by hand at night in the field. Specimens were generally located by listening for advertisement calls of males after dark. Whenever possible, calls were recorded to aid in identification to species level. Other specimens were collected opportunistically by searching along riverbanks, in overhanging vegetation along streams, on rocks in torrents, in reed beds and forest leaf litter. Specimens were euthanized with MS 222 (3-aminobenzoic acid ethyl ester or methanesulfonate salt) and fixed in 10% formaldehyde. After fixation, the specimens were cleared with water then transferred to 70% ethanol for long-term storage and preservation.

For histological detection of *Bd*, one whole foot of small specimens or 1–2 toes of larger specimens were surgically removed and decalcified in Perreni’s fixative for 18 h. Skin tissue was dehydrated further, elucidated with xylene, infiltrated with paraffin wax at 60°C (under vacuum) and embedded in paraffin wax blocks. Specimens were sectioned at 6 μm. Two slides containing two ribbons of 4–10 sections each were prepared for every specimen; Mayer’s haematoxylin was used as staining solution and eosin as counter stain. Slides were examined under a Nikon Eclipse E800 compound microscope for the presence of *Bd*.

For DNA detection of *Bd*, skin scrapes from samples were mixed by pipetting the liquid up and down, then the entire volume, including any visible skin/tissue pieces, was transferred to a micro-fuge tube. After spinning at maximum speed in a micro-centrifuge (~16 000*g*) for 3 min, the supernatant was drawn-off and discarded, tissue lysis buffer added and any pellet resuspended by vortexing. To this, 10 μg of carrier DNA was added to the lysis buffer. Total DNA was extracted from all samples using a spin-column DNA purification procedure.

Histopathology on a hind-foot was performed on a subset of *Bd*-positive animals. All developmental stages of *Bd* were observed in histological sections of infected animals. Usually, between one and three cell layers contained sporangia, and infected regions developed hyperkeratosis and hyperplasia (electronic supplementary material, figure S3). Infection was not restricted to any particular region on the toe, however, ventral surfaces, especially tubercles and toe discs, were frequently infected. No amphibian mortalities were recorded although most infected animals showed evidence of skin-sloughing that may have been associated with infection.

#### (ii) Survey no. 2, 2013–2015

Between November 2013 and March 2015, 60 adult and juvenile amphibians were swabbed for *Bd* in the Uzungwa Scarp Nature Forest Reserve, northeast of the Kihansi Gorge. Fine-tipped MW100 swabs (MWE, UK) were used to gently run the swab over the back, ventre and legs, ensuring the back legs and toes were well swabbed. All swabs were kept at environmental temperature (14–30°C) for the entire duration of field work. DNA was extracted directly from swabs in accordance with the *RACE* protocol [37] and were processed in a dedicated pre-PCR clean room in a decontaminated and UV sterilized cabinet. Swab tips were snapped-off and placed directly into Safe-Lock Eppendorf tubes containing 0.03–0.04 g of 0.5 mm silica homogenization beads and 60 μl of Prepman Ultra (ThermoFisher Scientific, MA, USA). Samples were bead-beated for 45 s at 30 Hz in a Qiagen TissueLyser II (Qiagen, Venlo, Netherlands) before being centrifuged at 14 500*g* for 30 s, a process that was repeated twice in total. Samples were then incubated at 100°C for 10 min, cooled for 2 min, then centrifuged again at 14 500*g* for 3 min. The supernatant was collected and stored at −80°C until used for lineage-specific qPCR [16].

## 3. Results

### (a) Epidemiology from metagenomics of archival mortalities

Evidence of *Bd* infection in Kihansi spray toads was first recorded during the terminal decline of the wild population (figure 1*a*) at which time histopathological examination detected severe burdens of infection that were consistent with those known to be lethal [20]. Accessing the last two preserved Kihansi spray toads that had been collected at the time of the 2003 population crash (electronic supplementary material, figure S2), we applied sequencing protocols developed for the analysis of ancient DNA to shotgun sequence *N. asperginis* skin biopsies in order to determine whether we could assemble the pathogen’s genome [38]. By combining our DNA reads with those from pre-existing *Bd* genomic datasets, we were able to successfully assemble complete 178 kbp *Bd* mitochondrial genomes and in doing so, demonstrate the feasibility of using archived specimens for molecular epidemiology.

Our genetic analyses found that the two Kihansi spray toads collected during the terminal population decline were infected with *Bd*CAPE, the endemic African lineage of the *Bd* [13,39]. This finding, coupled with previous histological and molecular diagnoses of chytridiomycosis [20] provide the first evidence of a *Bd*CAPE-associated extinction event in the wild. Initial exploratory analysis using a newly developed lineage specific diagnostic assay [16] found that the two Kihansi spray toad specimens showed triplicated qPCR amplification for the *Bd*CAPE lineage and no amplification for *Bd*GPL. Subsequently, we were able to generate enough *Bd* DNA reads from archived amphibian tissue samples to assemble the *Bd* mitochondrial genome (electronic supplementary material 3).

Phylogenetic analysis of 1205 mitochondrial variants revealed that the two Kihansi samples were genetically similar to one another, differing by only 46 SNPs. These genotypes were also closely related to previously sequenced isolates of *Bd*CAPE, clustering closely with two chytridiomycosis-associated isolates, DB8-4 (Drakensburg, South Africa) and TF5a1 (Torrent des Ferrerets, Mallorca). All five deeply diverged *Bd* lineages are represented in the phylogeny, with globally high bootstrap support and therefore confidence in the placement of AC040803_A and AC290703_A2 in the *Bd*CAPE lineage (figure 1*c*). In addition, a ML genetic clustering approach based on 724 variants, together with a principal component analysis, further supported the genetic relatedness of the two Kihansi samples with that of contemporary *Bd*CAPE isolates recently collected in southern Africa (figure 1). Wards clustering designated AC040803_A and AC290703_A2 as a member of the same cluster as other *Bd*CAPE isolates and a PCA partitions diversity of other *Bd*CAPE isolates away from the *Bd*GPL cluster (figure 1*d*).

### (b) Phylodynamic analysis charts a 20th-century expansion of virulent African *Bd*

The two genotypes infecting the Kihansi spray toads were closely related to previously sequenced *Bd*CAPE isolates, both known to cause amphibian chytridiomycosis or symptoms associated with chytridiomycosis. Isolate TF5a1 was collected from the Torrent de Ferrerets gorge during the Mallorcan midwife toad outbreak of chytridiomycosis, which was first witnessed in 2008 [10,40]. Isolate DB8-4 was collected atop the Drakensburg portion of the Great Escarpment bordering South Africa and Lesotho, from a population of Phofung river frogs (*Amietia hymenopus*) that has been anecdotally observed to show clinical signs of chytridiomycosis [41]. In light of these observations, and the histological evidence published by Weldon *et al*. [20], it would appear that the genotype found infecting the Kihansi spray toads was typical of *Bd*CAPE strains from southern Africa, and that are known to be virulent in nature.

Bayesian tip-dating was applied in BEAST2 [32], testing various plausible demographic and clock models until model convergence occurred (electronic supplementary material, table S3A). Our analysis supports a recent tMRCA of sampled diversity to between 14 and 67 years ago (mean of 26 years since the most recent sampling date), pointing to an African introduction of *BdCAPE* between 1944 and 2003, with tMRCA of 1991 (figure 2). Regardless of model specification, all estimates of posterior density encompass this time period (electronic supplementary material, figure S8A–D). Importantly, our estimated age of the common ancestor of African *Bd*CAPE suggests that the lineage invaded the Kihansi gorge region around the time that the hydropower dam was constructed.

### (c) Extinction-by-infection is limited to the Kihansi Gorge

To determine the extent of *Bd*CAPE’s occurrence and wider impact on the herpetofauna of the Udzungwa mountains, wider surveys of *Bd* across this landscape were performed. Here, we report two chytrid surveys of the Udzungwas: Survey no. 1, undertaken 2004–2005 (Weldon and Moyer); and Survey no. 2, undertaken 2013–2015 (Lyakurwa, Tonelli, Bowkett, Marsden).

#### (i) Survey no. 1, 2004–2005

**Figure 3.**
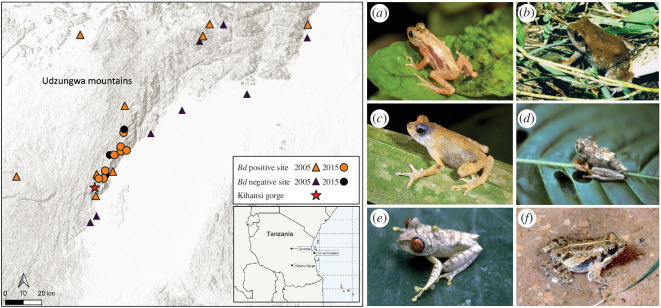
A map of the Udzungwa Mountain region in Tanzania outlining the locations of the histopathology/qPCR survey of 2005 and the qPCR swab survey of 2015. Positive swab sites are marked in orange and negative sites marked as black. Locations in the 2005 survey are marked by triangles, and the 3013–2015 survey in circles. The location of the Kihansi spray toad site is shown as a red star. Small insert map of Tanzania shows the location of the Kihansi Gorge relative to major cities. Photographs of the Kihansi spray toad and amphibian species that tested positive for *Bd*CAPE in 2003 and during the qPCR swab survey. (*a*) *N. asperginis,* (*b*) *Arthroleptis stenodactylus,* (*c*) *Nectophrynoides tornieri,* (*d*) *Hyperolius kihangensis,* (*e*) *Leptopelis parkeri,* (*f*) *Phrynobatrachus* sp—Photography credits: Robert C. Drewes; Martin Pickersgill; Stephen Zozaya; John V Lyakurwa; Elena Tonelli.

Specimens from 48 species were collected from 20 distinct localities in the Udzungwas across ~100 km (figure 3; electronic supplementary material, tables S4 and S5). For those species sampled, nine (45% of species) were positive for infection. The average prevalence of infection across all amphibian species from infected localities was 19.1% with a maximum prevalence of 63.4% recorded for Maganga Farm in the Mufindi District. Apart from this locality, infection levels were never higher than 23%. Combined *Bd* infection levels for lowland localities in Kilombero District was only 2%, whereas combined levels for highland localities was 14%. *Bd* was detected in 35 of the 554 (6.3%) specimens and 10 of 48 (8.7%) species screened in the test sample (electronic supplementary material, table S4). No lineage-specific qPCR was undertaken as the technique did not exist in 2005.

#### (ii) Survey no. 2, 2013–2015

Non-invasive skin swab samples were taken from 24 amphibian species across a distance of ~30 km in the Udzungwas, close to the Kihansi Gorge (figure 3). Of the 60 swab samples, 15 (25%) returned positives for *Bd*, with a GE count of between 0.01 and 71.1. These results echo those of the previous and more extensive 2005 *Bd* survey in the region that was completed just after the spray toad’s terminal population decline [20]. Of the five *Bd* positive samples that yielded a lineage-specific result, all were confirmed as *Bd*CAPE (electronic supplementary material, table S6). All amphibian species that were positive for *Bd* showed no signs of disease during the survey period. *Nectophrynoides tornieri* (Tornier’s viviparous toad; figure 3), a close relative of the Kihansi spray toad, was found to be infected by *Bd*CAPE infection, in comparison to the 2005 survey; despite infection the individuals displayed no evidence of clinical chytridiomycosis. However, we do note the absence of *N. poyntoni* in the surveys, a species which has not been observed since its discovery in 2003. While population viability analyses of these Udzungwa amphibian communities were not undertaken, no obvious signs of declines outside of the Kihansi Gorge were seen since the original 2005 survey and amphibians were readily found and sampled, including *N. tornieri*.

We note that owing to the different techniques used for surveillance between the two sampling periods, we cannot directly compare prevalence. However, as the studies span a decade, we can conclude with some certainty that the Udzungwa herpetofauna outside of the Kihansi Gorge coexist with *Bd*CAPE infection, with the caveat that systematic population viability analyses in future surveys are needed to formally show this.

## 4. Discussion

Our approach highlights the potential of using metagenomics twinned with phylodynamic analysis to explore the processes underlying population declines and extinctions of species by using specimens held in collections and museums. By deep sequencing archived *Bd*-infected Kihansi spray toads as they entered their terminal decline in the wild, we were able to refine our understanding of the epidemiology of this extinction-by-infection event by showing that *Bd*CAPE is spatially emerging in Africa and invaded the Udzungwa mountains around the time of the recorded *Bd* epizootic. This is the first record of an extinction event caused by *Bd*CAPE.

Our current understanding of the epidemiology and virulence of *Bd*CAPE is limited [40,42]. Experimental studies have shown that *Bd*CAPE can cause lethal chytridiomycosis and is more frequently associated with chytridiomycosis in wild species than is seen for *Bd*ASIA−1, *Bd*ASIA−2/BRAZIL or *Bd*ASIA3 [6,13]. Moreover, *Bd*CAPE led to a population decline following its introduction into the Balearic Island population of Mallorcan midwife toads (*Alytes muletensis*) [10,43]. Despite this, there remains a general perception that widely globalized *Bd*GPL is the only lineage of conservation concern. Although *Bd*GPL has been documented in many more declines than *Bd*CAPE, and has a much broader distribution [13], recent findings demonstrate that the *Bd*CAPE lineage is more widely distributed than previously thought [44]. Records of *Bd*CAPE from Europe and Central America are most likely to represent introductions from southern or western Africa where the majority of *Bd*CAPE observations have been made and where genetic diversity appears to be high [13,44].

Spatial surveillance and predictive ecological niche modelling of *Bd* in South Africa demonstrates that *Bd*GPL and *Bd*CAPE occupy different ecotypes, with the latter occurring across cooler higher altitude environments [45]. Our evidence of *Bd*CAPE causing lethal chytridiomycosis in the Udzungwa Kihansi spray toads expands the range of this lineage into a new area and highlights the threat posed to amphibian hosts by other, non-*Bd*GPL lineages [42,46].

The *N. asperginis* population was only discovered in 1996 and began to decline soon thereafter following construction of the hydropower dam [18,20,21]. While retrospective designation of disease as a primary driver of population declines or extinctions is highly challenging [7,47], the study by Weldon *et al*. [20] clearly identified *Bd* as the ultimate cause of the spray toads terminal decline. That we show other amphibian species including the related *N. tornieri* in the Udzungwas are infected with *Bd*CAPE yet show no evidence of disease, suggests that ecological change wrought by damming the Kihansi river may have amplified the impact of the invading pathogen. Parallel climate–disease interactions have been noted in other *Bd-*amphibian systems [3,11,48]. Importantly, that *Bd*CAPE is now known to occur widely in Africa can cause lethal amphibian chytridiomycosis and has now been associated with an amphibian extinction event, we clearly need to upgrade our assessment of the risk that this lineage poses to amphibian species more widely.

Widely across Africa, enigmatic amphibian species declines are increasingly linked to the spread of chytridiomycosis [42]. At the same time, range contractions of Sub-Saharan African amphibians have been detected for dozens of species [49]. What is lacking is integration across these efforts in order to better understand the extent that combinations of stressors that include climate change and habitat loss may exacerbate the impacts of infectious disease as a threatening process. Chytridiomycosis as a threat to Africa’s amphibians appeared inconsequential when compared against global patterns [7], however, our study emphasizes that emerging infections in wildlife disease are dynamic processes. Yet, policy actors, be they governmental or international, have not embraced the intersecting risk that environmental change and emerging diseases pose to species conservation. We argue here that risk assessments of large-scale habitat manipulations in environments that contain micro-endemic species urgently need to include the wider impact of multiple interacting stressors on these extremely vulnerable populations in order to better prioritize conservation actions.

## Data Availability

All sequences are available from NCBI BioSample accession: SAMN20599532. All datasets have been made available at [50]. Supplementary material is available online [51].
